# Over-diagnosed and over-treated: a survey of Australian public attitudes towards the acceptability of drug treatment for depression and ADHD

**DOI:** 10.1186/1471-244X-14-74

**Published:** 2014-03-13

**Authors:** Brad Partridge, Jayne Lucke, Wayne Hall

**Affiliations:** 1 UQ Centre for Clinical Research (UQCCR), The University of Queensland, RBWH Site, Herston, QLD 4029, Australia

**Keywords:** Attitudes, Depression, ADHD, Over-diagnosis, Medication, Anti-depressants, Stimulants

## Abstract

**Background:**

Over the last decade the use of psychotropic medications to treat common mental health problems has increased in Australia. This paper explores: 1) public attitudes towards the acceptability of using prescription drugs to treat depression and attention deficit hyperactivity disorder (ADHD), and 2) beliefs about over-diagnosis of depression and ADHD.

**Method:**

1293 members of the general public were surveyed about their attitudes towards drug treatment for depression and ADHD through the Queensland Social Survey (QSS), an omnibus state-wide survey of households in the state of Queensland. The survey was administered through a CATI (computer-assisted telephone interviewing) system. Logistic regression analyses were used to predict belief that drug treatment is acceptable, and that depression and ADHD are over-diagnosed.

**Results:**

Most participants (60.9%) said that it was acceptable to use prescription drugs to treat depression. In contrast, attitudes towards the use of prescription drugs to treat ADHD were much less positive with around the same proportion saying it was acceptable (42.1%) as unacceptable (38.2%). More than half of the sample agreed that too many people are diagnosed with depression when they don’t really have it (57.7%), and 78.3% of participants agreed that too many children are diagnosed with ADHD when they don’t really have it. Participants who said depression or ADHD were over-diagnosed were less likely to say that it is acceptable to treat these conditions with prescription drugs.

**Conclusions:**

Despite increases in prescribing rates there is still considerable scope for increasing the public’s acceptance of treating common mental illnesses with psychotropic drugs. Furthermore, the public’s views on over-diagnosis of depression and ADHD appear to reflect ongoing controversy about the proper identification of these conditions, and these views negatively impact attitudes towards drug treatment. This may be a barrier to effective treatment of these conditions given that drug treatment is often recommended as a first line response.

## Background

Over the last decade the use of psychotropic medications to treat common mental health problems has increased in Australia. In particular, increases in prescribing rates for anti-depressants and stimulants (typically used in the treatment of Attention Deficit Hyperactivity Disorder) were among the highest for all psychotropic drugs. A recent overview of Australian prescribing trends showed that prescribing rates rose 95% for antidepressants and 72% for ADHD drugs between 2000 and 2011 [1].

While it is clear that doctors are prescribing more psychotropic drugs, we know less about people’s attitudes towards drug treatment. Attitudes towards psychotropic drugs are an important part of the public’s “mental health literacy” [2,3] that may influence willingness to start using these drugs and continue to use them. The overall upward trend in the prescribing of psychotropic medications has been attributed in part to greater public awareness of mental illness (which may indicate increased treatment seeking on the part of patients) and a growing acceptance of psychotropic medications as an acceptable method of treatment for mental disorders [1]. Understanding public attitudes towards drug treatment is therefore an important part of understanding prescribing trends.

Public attitudes towards the use of anti-depressants have gradually become more favourable in Australia [4,5]. In 2011 we surveyed 1265 members of the Queensland public (as part of the Queensland Social Survey; QSS) finding that most people (55%) believed it was acceptable to use prescription drugs to treat depression; however, around 15% disagreed outright and almost 30% were unsure or neutral, indicating that there is considerable room for full acceptance of anti-depressants [6]. The two-thirds of participants had either been treated for depression with prescription drugs or knew someone who had, were more likely to view drug treatment as acceptable.

There is good evidence that public attitudes towards drug treatment are not uniform across mental disorders [7]. Our 2011 survey found that attitudes towards the use of stimulants to treat ADHD were sharply divided. The acceptability of drug treatment for ADHD was much lower than for depression (35%) and an almost equal number disagreed outright with such treatment [6]. While there is evidence that parents of children with ADHD are more likely to be accepting of stimulant treatment [8], other studies with Australian parents have found that those whose child had been diagnosed with ADHD are more likely to think that too many children are medicated for ADHD and two-thirds also thought that ADHD was over-diagnosed [9].

### Are attitudes towards over-diagnosis related to views about drug treatment?

Widening diagnostic criteria, off-label use drugs, and effective pharmaceutical industry marketing have also been suggested as factors contributing to the rise in psychotropic prescribing in Australia [1]. Some view this as evidence that increased prescribing is in fact the result of over-diagnosis and/or over-treatment of mental disorders [10]. Drug treatment is often the first line response to a diagnosis of depression or ADHD. However, the increase in stimulant prescribing in Australia has occurred amid ongoing worldwide controversy about the proper identification and diagnosis of ADHD in the first place [11,12].

Some studies have uncovered concerns that behaviours such as inattentiveness and excitability may be misattributed as symptoms of ADHD in some children (particularly boys), leading to suggestions that ADHD is over-diagnosed and stimulant drugs are being used without good cause [13]. A similar controversy exists about the over-use of antidepressants for depression [14-16]. While a number of health initiatives appear to have been successful at increasing the likelihood that sufferers of depression will seek and receive help [17], critics have suggested that increased prescribing of anti-depressants is often inappropriate and of questionable value [14].

The potential risks of unnecessary use of psychotropic medications include exposure to the side effects of drug treatment; a waste of financial resources on the part of the patient and the health care system; and potentially negative social effects of drug treatment, including stigma.

If the public believes mental illnesses are over-diagnosed and drug treatments are over-used as a result, then this may impact their attitudes towards the acceptability of treating these disorders with psychotropic medications. A belief that mental illnesses are over diagnosed and that drugs are overused may even deter some people from seeking help (which may not necessarily involve medication) even when they need it, thereby missing out on appropriate treatment. This may present an obstacle to effective treatment of mental disorders. There is a clear need to better understand public beliefs about over-diagnosis of mental disorders and their relationship to attitudes towards the acceptability of drug treatment of mental disorders.

### The current study

This study extends our 2011 study of public attitudes towards the acceptability of psychotropic drug treatment for ADHD and depression [6] by including an exploration of public attitudes towards over-diagnosis of these conditions. We again surveyed attitudes towards the acceptability of drug treatment for depression and ADHD given that: 1) anti-depressants and stimulants have increased more than almost all other psychotropic drugs, 2) there is ongoing discussion about over-diagnosis of both conditions, and 3) our previous study indicated relatively high levels of familiarity with both disorders among the general public.

Here we use data from the 2013 Queensland Social Survey. Our aims were to explore: 1) attitudes towards the acceptability of pharmacological treatment for depression and ADHD; 2) the extent to which attitudes were similar for the two disorders, 3) participants’ familiarity with people who have been treated for these two conditions using prescription drugs (e.g. have you or anyone you know personally been treated for depression/ADHD with prescription drugs?), and 4) the extent to which ‘familiarity’ is related to attitudes towards the acceptability of pharmacological treatment for depression and ADHD. These aims carry over from the 2011 study, allowing us to compare the 2011 and 2013 results. In addition, we aimed to explore: 4) public perceptions of over-diagnosis of depression and ADHD, and 5) the extent to which perceptions of over-diagnosis are related to attitudes towards the acceptability of drug treatment for these conditions.

## Methods

### Sample

The sample comprised 1293 participants: 671 males and 622 females (aged 18+ years; mean = 56.3). Older people were over-sampled when compared with the Australian Bureau of Statistics (ABS) census data for the Queensland population (those over 55 years of age were comprised 55.6% of the sample). Those aged under 35 years comprised 7.9% of the sample and those aged 35–54 years comprised 35.9%.

### The survey instrument: the Queensland social survey

Data collection was between June and August 2013 through the Queensland Social Survey (QSS), which is an omnibus style state-wide survey of households in the state of Queensland, Australia. The QSS is administered through a CATI (computer-assisted telephone interviewing) system at Central Queensland University (CQU). The CATI is implemented by a human interviewer who follows a computer guided series of survey questions from multiple research bodies and other organizations on a wide range of topics. Ethics approval for the study was granted by the CQU Human Research Ethics Committee. Participants were asked the extent to which they agreed with the following statements exploring attitudes towards the acceptability of drug treatment for depression and ADHD and their belief that these disorders are “over-diagnosed”:

Q1. It is acceptable for prescription drugs, such as Prozac, to be used in the treatment of depression.

Q2. Far too many people in Australia are diagnosed with depression when they don’t really have it. (Over-diagnosed)

Q3. It is acceptable for prescription drugs, such as Ritalin, to be used in the treatment of ADHD (Attention Deficit Hyperactivity Disorder).

Q4. Far too many children in Australia are diagnosed with ADHD when they don’t really have it. (Over-diagnosed)

Participants were asked to answer using the following response categories: (1) strongly agree; (2) agree; (3) slightly agree; (4) slightly disagree; (5) disagree; (6) strongly disagree; (7) don’t know. In Australia, Prozac and Ritalin are widely prescribed for the treatment of depression and ADHD, respectively, and are commonly mentioned in the media discourse about these conditions; we mentioned them by name to aid participant understanding.

Next, participants were asked about their familiarity with persons who have been treated for depression or ADHD with prescription drugs:

Q5. Have you, or someone you know personally, ever been treated for depression with prescription drugs?

Q6. Have you, or someone you know personally, ever been treated for ADHD with prescription drugs?

Participants were asked to answer according to the following format: (1) yes – I have; (2) yes – someone I know personally has; (3) yes – both myself and someone I know personally; (4) no; (5) don’t know.

### Procedure

The target population for the telephone interview consisted of persons 18 years of age or older who, at the time of the survey, were living in Queensland and could be contacted by direct-dialled, land-based telephone service. The response rate was 41.2%. The sample was drawn from a telephone database by using a computer program to select a random sample of phone numbers. Within the household, one eligible person was selected as the respondent for the interview whilst ensuring an equal yet random selection of male and female participants. Participants granted verbal consent to participate.

### Analysis

Descriptive analyses gave overall rates of: agreement with the acceptability of using prescription drugs to treat depression and ADHD; agreement that too many people/children are diagnosed with depression and ADHD; and overall rates of familiarity with drug treatment for depression and ADHD.

Two logistic regression analyses were conducted (one for depression and one for ADHD) to predict agreement with the acceptability of using prescription drugs to treat the disorder. The dependent variable was ‘It is acceptable to use prescription drugs’ coded as disagree = 0; agree = 1. The predictor variables were: gender (male = 0 (ref); female = 1); years of education (1-10 yrs = 0 (ref); 11-12 yrs = 1; 13-14 yrs = 2; 15 yrs + = 3); age (18–34 = 0; 35–44 = 1; 45–54 = 2; 55+ = 3); over-diagnosis (disagree = 0; agree = 1); and familiarity with the relevant disorder (not familiar = 0 (ref); yes – other = 1; yes – me = 2). For ADHD, the familiarity variable was coded as (not familiar = 0 (ref); yes – me or other = 1), due to the small number of participants who had actually been diagnosed with ADHD.

We also conducted two logistic regression analyses to predict agreement that 1) depression is over-diagnosed, and 2) ADHD is over-diagnosed. Predictor variables in these regression models were the demographic variables age, gender, education, and familiarity as described above.

## Results

Most participants (60.9%) said that it was acceptable to use prescription drugs to treat depression; only 17.5% expressed outright disagreement. In contrast, attitudes towards the use of prescription drugs to treat ADHD were much less positive with around the same proportion saying it was acceptable (42.1%) as unacceptable (38.2%). For both depression and ADHD, almost one-in-five participants answered “Don’t Know” (21.6% for depression and 19.6% for ADHD).

### Depression

1) *Over-diagnosis of depression*. More than half of the sample agreed that too many people are diagnosed with depression when they don’t really have it (57.7%), and 22.9% disagreed that this was the case (19.4% did not know). Of the participants who thought depression was over-diagnosed, 58.6% said drug treatment is acceptable. Those who did not think depression is over-diagnosed were more likely to agree that drug treatment is acceptable (77.3%) (see Table 1).

2) *Familiarity*. Two-thirds of the sample was familiar with someone who had been treated for depression with prescription drugs (67%) – either someone they personally knew (46.4%) or themselves (20.6%). The remainder (33%) did not know anyone who had been treated for depression with prescription drugs (“Not Familiar”). Fewer participants in the “Not Familiar” group believed that drug treatment was acceptable (51.4%) compared to those who did know someone who had been treated (63.2%), and those who had been treated themselves (70.8%). Almost a third of participants in the “Not Familiar” group answered “Don’t know” (30.3%) compared to participants who either knew someone who had been treated with drugs for depression (18%) or who had been treated themselves (14%).

3) *Predicting positive attitudes towards the acceptability of drug treatment for depression.* A logistic regression analysis was used to assess the impact of a number of factors on agreement with the proposition that it is acceptable to use prescription drugs to treat depression. The full model was able to distinguish between respondents who expressed outright agreement with the acceptability of using drugs to treat depression and those who did not [x^2^ (10, N = 844) = 61.584, p < .001]. Table 2 shows that participants who said that depression is over-diagnosed were 3.2 times less likely to think that drug treatment for depression is acceptable. Younger participants (18–34 years) were 2 times more likely than older participants (55 years +) to think that drug treatment for depression is Acceptable.

4) *Predicting the belief that depression is over-diagnosed*. People who were not familiar with anyone who had undergone drug treatment for depression were much more likely to think that depression is over-diagnosed [x^2^ (9, N = 1014) = 54.525, p < .001]. Participants with 1–10 years of education were more likely to think depression is over-diagnosed compared to those with 13–14 and 15+ years of education (see Table 3).

**Table 1:** Agreement with the statement ‘it is acceptable for prescription drugs to be used in the treatment of depression’ according to ‘familiarity’ and ‘attitudes towards over-diagnosis’

		**Acceptable for prescription drugs to treat depression**		
	**N (%)**	**Agree (%)**	**Disagree (%)**	**Don’t know (%)**
**Total sample**	1269	60.9	17.5	21.6
**Depression is over-diagnosed**				
Agree	732	58.6	24.0	17.3
Disagree	291	77.3	9.6	13.1
Don’t know	109	48.4	7.3	44.3
**Familiarity with depression**				
Not Familiar^a^	422	51.4	17.3	31.3
Yes – other^b^	593	63.2	18.7	18.0
Yes – me^c^	264	70.8	15.2	14.0
**Age category**				
18 – 34	101	74.3	13.9	11.9
35 – 44	199	70.4	10.1	19.6
45 – 54	262	67.2	14.5	18.3
55+	708	54.2	20.9	24.9

^a^Not familiar = I don’t know anyone who has been treated for depression with prescription drugs.

^b^Yes – other = I personally know someone who has been treated for depression with prescription drugs.

^c^Yes – me = I have been treated for depression with prescription drugs.

**Table 2:** Predictors of agreement with the statement: “it is acceptable for prescription drugs to be used in the treatment of depression”

**Predictor**	**Odds**	**95% CI**
**Over-diagnosed**		
Yes	.31***	.19 – .49
**Familiarity**		
Not familiar^a^	1	-
Yes - other^b^	1.16	.79 – 1.71
Yes - me^c^	1.53	.93 – 2.52
**Gender**		
Female	.93	.66 – 1.31
**Age (years)**		
18 – 34	1	-
35 – 44	1.22	.54 – 2.76
45 – 54	.71	.34 – 1.48
55+	.49*	.25 – .97
**Education**		
1 – 10 years	1	-
11 – 12 years	.95	.58 – 1.55
13 – 14 years	1.16	.66 – 2.01
15+ years	1.21	.79 – 1.87

*p<. 05; ***p < .001

^a^Not familiar = I don’t know anyone who has been treated for depression with prescription drugs.

^b^Yes – other = I personally know someone who has been treated for depression with prescription drugs.

^c^Yes – me = I have been treated for depression with prescription drugs.

**Table 3:** Predictors of the belief that depression is over-diagnosed

**Predictor**	**Odds**	**95% CI**
**Familiarity**		
Not familiar^a^	1	-
Yes - other^b^	.74	.52 – 1.04
Yes - me^c^	.43***	.28 – .64
**Gender**		
Female	.99	.74 – 1.31
**Age (years)**		
18 – 34	1	-
35 – 44	.85	.48 – 1.51
45 – 54	.87	.50 – 1.50
55+	1.21	.72 – .2.03
**Education**		
1 – 10 years	1	-
11 – 12 years	.68	.43 – 1.08
13 – 14 years	.57*	.35 – .94
15+ years	.41***	.27– .61

*p<. 05; ***p < .001.

^a^Not familiar = I don’t know anyone who has been treated for depression with prescription drugs.

^b^Yes – other = I personally know someone who has been treated for depression with prescription drugs.

^c^Yes – me = I have been treated for depression with prescription drugs.

### ADHD

1) *Over-diagnosis of ADHD*. The overwhelming majority of participants agreed that too many children are diagnosed with ADHD when they don’t really have it (78.3%), and only 8.4% of participants disagreed. Among those who said ADHD is over-diagnosed, 41.5% said drug treatment for ADHD is acceptable, whereas 59.4% of participants who did not think ADHD is over-diagnosed said drug treatment is acceptable (see Table 4).

2) *Familiarity*. Most participants (61.1%) did not know anyone who had been treated for ADHD with prescription drugs (“Not Familiar”). 35.3% knew someone who had been treated, and only 3.7% had been treated themselves. The ratio of agree/disagree responses was slightly higher for the two “Familiar” groups compared to those who did not know anyone treated for ADHD with drugs. Those in the “Not Familiar” group had the lowest overall rate of agreement with the acceptability of drug treatment for ADHD (36.5%), however a much higher proportion of people in the “Not Familiar” group also answered “Don’t know” (27.1%), compared to the two “Familiar” groups.

3) *Predicting positive attitudes towards the acceptability of drug treatment for ADHD*. For ADHD, the full regression model was also statistically significant and able to distinguish between respondents who expressed outright agreement, and those who disagreed, with the use of drugs to treat ADHD, [x^2^ (8, N = 930) = 46.489, p < .001]. Table 5 shows that familiarity, over-diagnosis, education and age were significant predictors, while gender was not. Compared to participants in the “Not Familiar” group, those who knew someone who had received drug treatment for ADHD (or had themselves been treated) were 1.3 times more likely to agree that drug treatment is acceptable. As with depression, the belief that ADHD is over-diagnosed was associated with less favourable attitudes towards the acceptability of drug treatment. In addition, participants with 15+ years of education were 1.75 times more likely to agree that drug treatment for ADHD is acceptable compared to those with 1–10 years of education. As with depression, younger participants (18–34 years) were more likely than older participants (55+) to say that drug treatment for ADHD is acceptable. The regression model predicting the belief that ADHD is over-diagnosed was not significant, indicating that demographic variables (including familiarity) were not directly related to attitudes towards over-diagnosis, [x^2^ (8, N = 1089) = 6.529, p = .588] (see Table 6).

**Table 4:** Agreement with the statement ‘it is acceptable for prescription drugs to be used in the treatment of ADHD’ according to ‘familiarity’ and ‘attitudes towards over-diagnosis’

		**Acceptable for prescription drugs to treat ADHD**		
	**N (%)**	**Agree (%)**	**Disagree (%)**	**Don’t know (%)**
**Total sample**	1276	42.1	38.2	19.7
**ADHD is over-diagnosed**				
Agree	993	41.5	44.0	14.5
Disagree	106	59.4	31.1	9.4
Don’t know	169	34.3	9.5	56.2
**Familiarity with ADHD**				
Not familiar^a^	779	36.5	36.5	27.1
Yes – other^b^	450	50.4	40.7	8.9
Yes – me^c^	47	55.3	42.6	2.1
**Age category**				
18 – 34	101	56.4	33.7	9.9
35 – 44	198	41.9	37.9	20.2
45 – 54	260	52.3	35.0	12.7
55+	708	36.2	40.1	23.7

^a^Not familiar = I don’t know anyone who has been treated for ADHD with prescription drugs.

^b^Yes – other = I personally know someone who has been treated for ADHD with prescription drugs.

^c^Yes – me = I have been treated for ADHD with prescription drugs.

**Table 5:** Predictors of agreement with the statement: “it is acceptable for prescription drugs to be used in the treatment of ADHD”

**Predictor**	**Odds**	**95% CI**
**Over-diagnosed**		
Yes	.53**	.34 – .83
**Familiarity**		
Not familiar^a^	1	-
Yes - other^b^	^d^1.33*	1.02 – 1.74
Yes - me^c^		
**Gender**		
Female	1.03	.79 – 1.35
**Age (years)**		
18 – 34	1	-
35 – 44	.59*	.34 – 1.04
45 – 54	.82	.49 – 1.39
55+	.54*	.33 – .88
**Education**		
1 – 10 years	1	-
11 – 12 years	1.1	.73 – 1.64
13 – 14 years	.83	.53 – 1.31
15+ years	1.75**	1.23 – 2.48

*p<. 05; **p < .01.

^a^Not familiar = I don’t know anyone who has been treated for ADHD with prescription drugs.

^b^Yes – other = I personally know someone who has been treated for ADHD with prescription drugs.

^c^Yes – me = I have been treated for ADHD with prescription drugs.

^d^Includes participants who have been treated for ADHD with prescription drugs or who know someone who has.

**Table 6:** Predictors of the belief that ADHD is over-diagnosed

**Predictor**	**Odds**	**95% CI**
**Familiarity**		
Not familiar^a^	1	-
Yes - other^b^	^d^.76	.50 – 1.14
Yes - me^c^		
**Gender**		
Female	.90	.59 – 1.36
**Age (years)**		
18 – 34	1	-
35 – 44	.93	.42 – 2.03
45 – 54	1.28	.59 – 2.78
55+	1.47	.72 – 3.01
**Education**		
1 – 10 years	1	-
11 – 12 years	1.02	.54 – 1.91
13 – 14 years	1.25	.60 – 2.59
15+ years	.93	.54 – 1.61

^a^Not familiar = I don’t know anyone who has been treated for ADHD with prescription drugs.

^b^Yes – other = I personally know someone who has been treated for ADHD with prescription drugs.

^c^Yes – me = I have been treated for ADHD with prescription drugs.

^d^Includes participants who have been treated for ADHD with prescription drugs or who know someone who has.

## Discussion

Prescriptions for anti-depressants and stimulants in Australia have increased at a higher rate than almost all other psychotropic medications over the last decade [1,18,19]. However, there are ongoing concerns about the extent to which this increased prescribing is accepted by the public, has led to better health outcomes, or is symptomatic of over-diagnosis of mental disorders [12].

This study found that drug treatment for depression is acceptable to most members of the public (approx. 60%), although almost 40% disagreed or were unsure. This result echoes our 2011 findings (see Table 7) [6]. In Australia and the US, public views about the use of anti-depressants have become more positive since the 1990s [4,7]. In terms of overall support, some surveys suggest a higher level of endorsement for anti-depressants in the treatment of depression among members of the US public compared to the participants in our survey [7]. Drug treatment for depression was regarded as much more acceptable than drug treatment for ADHD. Overall, members of the public were ambivalent about the acceptability of using prescription stimulants to treat ADHD, and only 42% agreed that drug treatment is acceptable for ADHD. This is in line with our 2011 findings and with US surveys showing that a minority of members of the public would choose stimulant medication for a child, and most would prefer behavioural interventions [20,21].

**Table 7:** Reponses to the statements “it is acceptable to use prescription drugs to treat Depression/ADHD”: 2013 and 2011 results

	**Depression (%)**		**ADHD (%)**	
	**2013**	**2011**^**a**^	**2013**	**2011**^**a**^
**Agree**	60.9	61.4	42.1	39.2
**Disagree**	17.5	16.7	38.2	39.3
**Don’t Know**	21.6	21.9	19.6	21.5

^a^2011 data included a “Neutral” option accounting for 9.6% of the sample for depression, and 9.2% for ADHD. The percentages reported here have been re-calculated by excluding “Neutral” respondents from the denominator for direct comparison with 2013 data.

Our exploration of attitudes towards over-diagnosis yielded results that health care professionals may find concerning – that is, most participants believed that depression and ADHD are over-diagnosed in the first place. In particular, around 78% of participants said that too many children are diagnosed with ADHD when they don’t actually have the disorder. And despite most participants saying that drug treatment for depression is acceptable, nearly 58% said that too many people are diagnosed with depression when they don’t really have it. Importantly, people who thought that depression or ADHD were over-diagnosed were less likely to think that it is acceptable to use prescription drugs to treat these disorders. This raises the prospect that scepticism about drug treatment may be partly based on scepticism about the appropriateness of the diagnosis. Some members of the public who believe that depression and ADHD are too readily diagnosed may be reluctant to seek help from a health care provider even when they (or their children) need it. This may be for a number of reasons including concerns about unnecessary drug treatment and fear of being stigmatised by having a mental health problem. In such cases, perceptions about over-diagnosis and over-treatment could in fact result in some people in genuine need for help going untreated. One other potential implication is that members of the public who do receive a diagnosis of depression or ADHD may be more reluctant to initiate or continue drug treatments if they believe these conditions are typically over-diagnosed and over-medicated. This may be a barrier to effective treatment of these conditions given that drug treatment is often recommended as a first line response. It may be worthwhile for future research to explore how perceptions about diagnosis and treatment influence actual treatment seeking behaviour. For ADHD especially, it would be useful for future studies to explore in greater detail how attitudes towards stimulant prescribing are related to attitudes towards the diagnosis of ADHD, and to the use of stimulant medication. Parents of a child with ADHD may have concerns about the side-effects of stimulant treatment however these concerns may be amplified if they harbour doubts about the proper diagnosis of ADHD.

Members of the Australian public in this study showed high levels of familiarity with drug treatment both conditions – around 67% of participants had either been treated for depression with prescription drugs or personally knew someone who had (65.4% in 2011). Approximately 35% knew someone who had been treated for ADHD with stimulants (28.7% in 2011). Our exploration of other factors predicting positive attitudes towards the acceptability of drug treatment yielded slightly different results to 2011. Knowing someone who had been treated for depression with prescription drugs (or having been treated oneself) was not a significant predictor of positive attitudes towards the acceptability of drug treatment for depression – unlike our 2011 survey. While our 2013 results showed a clear trend towards familiarity being a predictor, it did not reach statistical significance. Other studies with members of the Australian public have suggested that familiarity may be associated with more favourable attitudes towards drug treatment. For example there is evidence that Australians who know someone with depression are less likely to think anti-depressants are harmful [22]. Studies in other countries have found similar results [23]. For ADHD, familiarity was a significant predictor of more positive attitudes towards the acceptability of drug treatment in the current study – the 2011 analysis did not reach significance. This is again an area that well designed qualitative studies could help to elucidate the nature of the relationship between these variables. While familiarity may lend a greater insight into the potential benefits of drug treatment, those who personally know a child treated for ADHD with prescription drugs are also likely to be acutely aware of any negative side-effects.

Although age was not a significant predictor of attitudes towards over-diagnosis, older people were much less likely to say that drug treatment for depression or ADHD is acceptable. This finding warrants further exploration given that older people were over-represented in this sample and participants over 55 years of age were more likely to answer “don’t know”. Compared to all other age categories, those aged 18–34 were far less likely to answer “don’t know”.

Attitudinal surveys can provide a useful snapshot of the public viewpoints. The usefulness and robustness of their findings can be increased if they are repeated. Although there has been an increase in the prescription of drugs to treat depression and ADHD, the findings from the 2013 QSS and 2011 QSS indicate that there is still considerable scope for convincing many members of the public that drug treatment is acceptable or warranted. Addressing the belief that these disorders are over-diagnosed appears to be one part of this. This survey could be conducted again in the future to essentially track attitudes towards these issues over time and further elucidate the factors that influence attitudes. The major limitation of using the sample described in this study is that younger people are under-represented. While this is not unusual for CATI surveys with the general community, it does mean that it would be prudent not to overgeneralise these findings. Under representation of younger people often occurs in CATI surveys that source landline phones only, and dual frame (landline and mobile) surveys may improve the representativeness of future surveys. Nevertheless the survey tool could easily be used with other representative cohorts for direct comparisons or extended to explore other domains relevant to public attitudes towards the acceptability of drug treatment (as we did in the 2013 survey by extending the analyses to explore “over-diagnosis”). This would potentially improve the explanatory power of the survey tool by taking into account a larger number of variables that inform attitudes. The survey could also be adapted to investigate attitudes towards the acceptability of drug treatment for other common mental illnesses such as anxiety and various substance use disorders. This would provide a broader understanding of the spectrum of public attitudes towards pharmacological drug treatment. Elsewhere, we have used a variation of this survey to explore attitudes towards the acceptability of healthy people using prescription drugs for “cognitive enhancement” [24].

## Conclusions

This paper demonstrates the importance of understanding public attitudes towards treatments for disorders such as depression and ADHD. Most members of the public thought depression and ADHD were over-diagnosed, although they were more accepting of drug treatment for depression compared to ADHD. Increases in the prescription of psychotropic medication may not result in better treatment if there are widespread public attitudes that do not support the use of drug treatment for these conditions. Further research about public attitudes may provide helpful insights into ways in which treatment for depression and ADHD may be better managed. In particular, it would be worthwhile investigating what members of the public and/or health care professionals think should be done to avoid over-diagnosis (for example, requiring parent *and* teacher data to inform an ADHD diagnosis, or restricting the diagnosis to major types of impairment) and to avoid overtreatment (for example, trying cognitive behavioural therapy for depression before prescribing antidepressants).

## Competing interests

This research was supported by a University of Queensland internal staff research grant awarded to Brad Partridge. Brad Partridge has been awarded an NHMRC post-doctoral fellowship. Wayne Hall has been awarded an NHMRC Australia Fellowship.

## Authors’ contribution

BP, JL, and WH contributed to the design of the study and creation of the survey items. BP analysed the data. BP, JL, and WH contributed to the drafting of the manuscript. All authors read and approved the final manuscript.

## Pre-publication history

The pre-publication history for this paper can be accessed here:

http://www.biomedcentral.com/1471-244X/14/74/prepub
